# Letter to the Editor

**DOI:** 10.1186/1617-9625-3-1-41

**Published:** 2005-12-15

**Authors:** A Zavras, M Falagas

## 

Chair of Scientific Committee:

Dr. Athanasios I. Zavras, Harvard University – ISPTID President

Dr. Matthew Falagas, Tufts Univ. & Dunant Hospital

Scientific Committee Members:

Dr. Cynthia Boschi, World Health Organization

Dr. Carolyn Dresler, International Agency for Research on Cancer, WHO

Prof. Anthony Hedley, University of Hong Kong School of Medicine

Asst. Prof. Taru Kinnunen Mustonen, Harvard University School of Dentistry

Prof. Steve Sussman, Univ. of California San Francisco

Prof. David Scott, Univ. of Louisville School of Dental Medicine

Dr. Alexandra Shields, Georgetown University Institute for Health Policy

Organizing Committee Members:

Prof. Konstantinos Alexandridis, Univ. of Athens School of Dental Medicine

Asst. Prof. Vassillis Gorgoulis, Univ. of Athens School of Medicine

Dr. Argyris Michalopoulos, Intensive Care Unit, Dunant Hospital

Dr. Katerina Katsampe, Deputy Mayor of Athens

Prof. John Kyriopoulos, National School of Public Health

Mr. Kyriakos Souliotis, Onasion Cardiothoracic Surgery Hospital

Mr. Dimitrios Zavras, National School of Public Health

Dear Delegates

It is a great honor to host the 4th annual meeting of the International Society for the Prevention of Tobacco Induced Diseases in Athens, Greece. As the birthplace of democracy, Athens seems the ideal place for debate. And the subject, quantifying tobacco's impact, seems most suitable for scientific exchange.

Tobacco use is the single most important preventable risk factor for a number of debilitating diseases around the world. ISPTID global; membership is dedicated to the prevention of tobacco induced diseases. Some of the primary aims of the annual ISPTID conference are a) to disseminate important, state-of-the-science information about the biologic effects and the public health impact of tobacco use, b) to present updated data about the clinical signs and symptoms of tobacco induced disease and the various methods of smoking control and prevention, c) to create and facilitate research networks, and d) to educate healthcare professionals in tobacco control.

Financial interests, trade issues and taxation have allowed tobacco's spread for decades. The international community has recently agreed on a number of measures to curb tobacco use by adopting the first ever public health treaty, the Framework Convention of Tobacco Control (FCTC). The first indications of FCTC success around the world will be presented at the conference.

Apart from their strong international presentations, ISPTID conferences attempt to contribute to the promotion of tobacco control locally. Given the high prevalence of smoking in Greece, the Society will address the local media and will host a workshop for healthcare professionals on how to design and implement tobacco cessation programs in their prospective clinical settings.

We hope you enjoy the meeting.

For better health,

Athanasios I. Zavras, DMD, MS, DrMsc

ISPTID President

Matthew Falagas, MD, MPH

Chair of the Scientific Committee

## Competing interests

The authors declare that they have no competing interests.

## Supplementary Material

Additional file 1 Please see additional file 1 Click here for file

