# Supplementary material for: Letter to the Editor
Source: Tob Induc Dis. 2005 Dec 15;3(1):41. doi: 10.1186/1617-9625-3-1-41 (PMC2643412; doi:10.1186/1617-9625-3-1-41)
Supplement: Additional file 1 — Please see additional file 1 [file 1617-9625-3-6-S1.pdf]

PTID 2005 Scientific Program

September 30th to October 2nd, 2005

Friday Sept. 30th

Registration Booth opens at 7:30am

Morning Session 9:00-12:00pm Basic Science

Time	Title	Author(s)
9:00-9:05am	Welcome Address	* Athanasios I. Zavras<br>Harvard University, Boston, Ma. USA
9:05-9:30am	Keynote Address<br>Cigarette smoking and innate immunity	* David Scott<br>MODERATOR<br>University of Louisville, KY, USA
9:30-9:45am	Cigarette smoking and endothelial cell injuries	* Xing Li Wang<br>Baylor College of Medicine, USA
9:45-10:00am	Cigarette smoking and endothelial cell injuries	* David Bernhard<br>Medizinische Universitat Innsbruck, Austria
10:00-10:15am	Ethnic differences in nicotine metabolic rate and genes in New Zealand	* Lea A Rod, N Benowitz, M Green, M Lea, A Woodward, J Fowles, G Chambers, D Phillips<br>Institute of Environmental Science & Research, New Zealand
10:15-10:30am	Nicotine and cotinine alter the metabolism of surfactant-producing fetal rat lung type II alveolar cells	* Merluza, J.A., Hillier, C., Thliveris, J.A., Halayko, A., Shaw, A.R., Sowa, M. Scott, D.A. and Scott, J.E.<br>Univ. of Manitoba, Natioanl Research Council, Manitoba Institute of Child Health, Winnipeg, Canada.
10:30-11:00am	COFFEE BREAK	
11:00-11:15am	Mitogen Activated Protein Kinase (MAPK) Signaling by Nicotine in Primary and Pancreatic Tumor cells: Effects on Proliferation and Cell Function.	*Parimal Chowdhury, Kodetthoor B. Udupa & Vladimir P. Zharov.<br>Central Arkansas Veterans Health Care System, Little Rock, Arkansas, USA and University of Arkansas for Medical Sciences, College of Medicine, USA
11:15-11:30am	Identification of molecular alterations in periodontal tissues using infrared micro-spectroscopy	* Kan-Zhi Liu, Man Angela, Singer David L, Sowa Michael G., David A Scott<br>National Research Council Canada, University of Manitoba, Winnipeg, Canada
11:30-11:45am	Expression of the alpha7-nicotinic receptor in surfactant-producing fetal lung type II cells and fibroblasts	* Elliott Scott<br>MODERATOR<br>University of Manitoba, Winnipeg, Canada
11:45-12:00pm	Functional characterization of Syrian hamsters congenic at the NAT2 locus for the N-acetylation of the tobacco carcinogen 4 -aminobiphenyl and the O-acetylation of its N-hydroxy-metabolite	* David W. Hein and Mark A. Doll<br>University of Louisville School of Medicine, Louisville, KY USA
12:00-1:00	LUNCH BREAK	

Friday Sept. 30th

Afternoon Session **1:00-5:00PM** **Tobacco Cessation / Control**

Time	Title	Author(s)
1:00: 1:15pm	Smoking in the elderly: the preventable fraction	* Tai Hing Lam<br>Univ. of Hong Kong
1:15-1:30am	Factors protecting women who quit smoking during pregnancy from smoking relapse after delivery Polish study.	* W. Hanke, K. Polanska, J. Lowe<br>Nofer Institute of Occupational Medicine, Lodz, Poland
1:30-1:45pm	Tobacco cessation efforts in Women	* Taru Kinunen<br>MODERATOR<br>Harvard University School of Dentistry, USA
1:45-2:00pm	Tobacco prevention & immigrant groups	* Per Haglind<br>Environmental Administration, City of Gotemborg, Sweden
2:00-2:15pm	Community resources to prevent or reduce addiction. The experience from Athens.	* Katerina Katsabe<br>Athina Hygeia Center for Prevention of Addiction, GR
2:15-2:30pm	Secondhand smoke exposure from friends & restaurants & respiratory symptoms in never-smoking adolescents	* Lai MK, Ho SY, Lam TH<br>University of Hong Kong, China
2:30-2:45pm	Empowering youth in tobacco control efforts in Malaysia	* Razak Lajis<br>National Poison Centre, University of Malaysia
2:45-3:00pm	Project EX: An example of a systematically developed teen tobacco use cessation program	* Steve Sussman<br>University of Southern California, USA
3:00-3:30pm	COFFEE BREAK	
3:30-3:45pm	Smoking habits, knowledge and attitudes among hospital staff in Tunisia	* Fakhfakh R, Boujemaa O, Ben Salah F, Gharbi R, Klouze A , Lakhal M, Belkahia C, Achour N<br>National Institute of Health, Hopital Charles Nicolle, and Centre National de Pharmacovigilance, Tunisia
3:45-4:00pm	Healthcare workers & patients' willingness to participate in and pay for smoking cessation	B. Finegan, T.C. Wild, * R. Sachs, D. Gordon<br>University of Alberta, Canada
4:00-4:15pm	Research perspectives on the nurse practitioner's role in smoking cessation therapy.	* Dr Sophia Chan<br>MODERATOR<br>University of Hong Kong, HK
4:15-4:30pm	Smoking Cessation in the Pre-Surgical Clinic: The Reduce My Risk Program	* B. Finegan, T.C. Wild, R. Sachs, C. Pysyk, U. Jakabos, & Y. Tul<br>University of Alberta, Canada
4:30-4:45pm	Pre-quitting nicotine replacement therapy is well tolerated, not associated with toxicity and acceptable to intending quitters at a cessation clinic	* Chris Bullen, Robyn Whittaker, Natalie Walker, Mark Wallace-Bell<br>University of Auckland, and Christchurch School of Medicine, University of Otago, Christchurch, New Zealand.
4:45-5:00pm	Acupuncture in smoking cessation	V. K. Agarwal*, S. Agarwal<br>D. D. U. Hospital, New Delhi, India

Saturday Oct 1st

Morning Session 9:00 12:00pm Topics of Clinical Importance

Time	Title	Author(s)
9:00-9:05am	Smoking and cardiopulmonary diseases	* D. Stamatelopoulos<br>MODERATOR<br>Univ.of Athens School of Medicine, Athens GR
9:05-9:20am	Sudden cardiac death and its association with tobacco use	* Xenophon Costeas<br>Henry Dunant Hospital, Athens GR
9:20-9:40am	Smoking and COPD	* Argyris Michalopoulos<br>Henry Dunant Hospital, Athens GR
9:40-10:00am	Tobacco use and lung diseases in women	* Dora Orfanidou<br>Univ.of Athens School of Medicine, Athens GR
10:00-10:20am	Smoking cessation and the lungs	* Michael Toubis<br>Sotiria Hospital for Chest Diseases, Athens GR
10:20-10:40am	COFFEE BREAK	
10:40-10:45am	The multiple faces of smoking related morbidity	* Matthew Falagas<br>MODERATOR<br>Alfa Institute for Biomedical Sciences, Athens GR
10:45-11:00am	Smoking and the metabolic syndrome	* Neil Thomas<br>University of Hong Kong, HK
11:00-11:20am	Smoking & Pregnancy	* George Daskalakis<br>University of Athens School of Medicine, GR
11:20-11:40am	Smoking and infection	* Antonios Vassiloyanakopoulos<br>Henry Dunant Hospital, Athens GR
11:40-12:00pm	Smoking and periodontal diseases	* Marizenia Pepelasi, Spyros Silvestros<br>Univ. of Athens School of Dentistry, Athens GR
12:00-1:00pm	LUNCH BREAK	

Afternoon Sessions 1:00 - 5:00pm Topics of Clinical Importance

Time	Title	Author(s)
1:00-1:05pm	Session on Smoking and Cancer	* Constantinos Alexandridis<br>MODERATOR<br>Univ. of Athens School of Dentistry, Athens GR
1:05-1:20pm	Tobacco use and cancer	* Christos Christodoulou<br>Henry Dunant Hospital, Athens GR
1:20-1:40pm	Importance of Smoking Cessation in Cancer Patients.	* Carolyn Dresler<br>Tobacco and Cancer Group, International Agency for Research on Cancer - Lyon, France
1:40-2:00pm	Is smoking the principal risk factor for urinary bladder cancer?	*Baena AV, Allam MF, Diaz-Molina C, Del Castillo AS, Adbel-Rahman AG, Navajas RFC<br>University of Cordoba, Spain
2:00-2:20pm	Smoking and oral cancer: knowledge of risk factor status among Navy recruits in Greece.	*Anastasios Mamalis, A.I. Zavras, S. Silvestros<br>University of Athens School of Medicine, GR
2:20-2:40pm	Toluidine Blue staining and Brush Biopsy in the early diagnosis of tobacco related oral lesions	* Mehrotra R, Singh M, Gupta A.<br>Univ. of Allahabad Medical College, India.
2:40-3:00pm	COFFEE BREAK	

Saturday Afternoon Sessions (cont.) 1:00 - 5:00pm Topics of Clinical Importance

3:00 - 4:00pm Tobacco Cessation Workshop for Healthcare Professionals

3:00-3:05pm **WORKSHOP**
Smoking cessation for healthcare professionals

* Catherine Hayes
MODERATOR
Harvard University, USA

In various parts of the world, medical doctors and dentists have incorporated tobacco smoking cessation in the services they offer to smokers.

The purpose of the Workshop is to empower clinicians to use tobacco cessation in their everyday practice.

The sessions will a) present the social framework of a tobacco cessation program and b) address the various steps of a tobacco cessation program in a systematic and practical way.

At the end of the workshop, participants will be able to understand issues of social marketing and message delivery, as well as design and implement a tobacco cessation practice.

3:05-3:20pm Social marketing of smoking cessation

* Nadia Dima
Athina Hygeia Center for Prevention of Addiction, Athens GR

3:20-4:00pm A program for smoking cessation in the Clinic

* Catherine Hayes
Harvard University, USA

4:00-7:00pm Acropolis & the Parthenon: Lecture & Site seeing in Athens

Sunday Oct 2nd

Executive Committee Business Meeting 9:00 - 10:30am (Open to ISPTID members)
TID Editorial Board Meeting 10:30 - 11:30am (Open to TID Editors & Board members)

Afternoon Session 1:00 - 5:30pm - Health Policy, Law, Economics & FCTC

Time	Title	Author(s)
1:00: 1:20pm	Building a model of the social costs of tobacco	* Dr. Sarah McGhee<br>MODERATOR<br>Univ. of Hong Kong, HK
11:20-1:40am	The true cost of tobacco usage in the US: health costs, taxes, productivity loss and tobacco grants.	* Denis F Kinane, John N Williams<br>Univ. of Louisville, KY, USA
1:40-2:00pm	Application of a Behavioral Ecological Model to guide policy and practice for population-level tobacco control	* Mel Hovell, Stergios Roussos, Ana P. Martinez-Donate, Carol Sipan, Ming Ji, and Newell Johnson.<br>Graduate School of Public Health, San Diego State Univ.
2:00-2:20pm	Application of a Behavioral Ecological Model to study cultural influences on disparities in clinician anti-tobacco counseling	*Stergios Roussos, Mel Hovell, Linda Hoskins, and Ana P. Martinez-Donate<br>Graduate School of Public Health, San Diego State Univ.
2:20-2:40pm	Sustaining anti-tobacco cultures: Mission impossible in Turkey?	*Dr. Sibel Gogan<br>School of Public Health, Ankara, Turkey
2:40-3:00pm	Applications and implications for tobacco control research and intervention in Greece	* Anthony Kafatos<br>University of Crete School of Medicine, Greece
3:00-3:20pm	COFFEE BREAK	
3:20-3:25pm	Tobacco Control & Health Policy: An introduction	* Dr. Athanasios Zavras<br>MODERATOR<br>Harvard University, USA
3:25-3:40pm	Challenges to tobacco control in the community: race, genetics, health professionals & health policy	* Alexandra Shields<br>Georgetown Institute for Health Policy, Washington, D.C, USA
3:40-4:00pm	Policy & the culture of smoking	* Barbara Kondilis<br>Hellenic American University, Athens GR
4:00-4:20pm	Challenges of prevention of environmental tobacco smoke in the workplace in Taiwan	Taipau Chia, YF Wang, JY Chiou, WL Yu, YS Huang<br>Univ. of Taiwan
4:20-4:40pm	The impact of the Health Promotion Law in Japan on the network of public health centers	* S. Iwanaga, T. Nakahara, K. Satomura, M. Noami, R. Sakamoto<br>Faculty of Medicine, Kyoto University, Japan
4:40-5:00pm	Social change after the Health Promotion Law and FCTC in Japan	*Kazunari Satomura, Toshitaka Nakahara, Suketaka Iwaanaga, Ryota Sakamoto, Megumi Noami, Toru Takahashi.<br>Dept. of Public Health, Faculty of Medicine, Kyoto University, Japan
5:00-5:20pm	Towards comprehensive tobacco control; the political process and public health advocacy in Hong Kong	*Prof Anthony Hedley<br>University of Hong Kong, HK
5:20-5:30pm	Closing of The 4th Annual Conference<br>5th ANNUAL ISPTID CONFERENCE IN HONG KONG	* Professor Anthony Hedley
